# Editorial: Socioeconomic inequalities in digital health

**DOI:** 10.3389/fdgth.2025.1680350

**Published:** 2025-11-27

**Authors:** Lua Perimal-Lewis, Sónia Vladimira Correia, Evanthia Sakellari

**Affiliations:** 1 Flinders University, Adelaide, SA, Australia; 2 Univerzita Palackeho v Olomouci, Olomouc, Czechia; 3 Universidade Lusófona de Lisboa, Lisboa, Portugal; 4 University of West Attica, Athens, Greece

**Keywords:** digital health, socioeconomic inequalities and health, socioeconomic inequalities in digital health, universal health coverage (UHC), digital exclusion, digital readiness, digital marginalisation, digital literacy

Socioeconomic inequalities - rooted in disparities in income, education, and access to resources, continue to shape individuals' life chances and health outcomes across generations (1, 2). Nowhere is this more evident than in healthcare, where structural barriers systematically disadvantage low-income and marginalised communities, contributing to preventable illness and early mortality (3). As healthcare systems around the world increasingly adopt digital technologies, these longstanding disparities are taking on new forms. What promises to democratize care could, without equity, become a driver of deeper exclusion.

Socioeconomic inequalities in digital health refer to the uneven access, use, and benefit from technologies such as telemedicine, mobile health apps, and wearable devices based on social and economic status. While these tools have the potential to enhance care, improve efficiency, and reach underserved populations, they may also exacerbate disparities if barriers such as digital literacy, internet access, affordability, and trust remain unaddressed (4, 5). Ensuring that digital health promotes collective well-being requires us to see beyond technical efficiency and ask more fundamental questions about ethics, equity, and purpose. Justice implies equitable access to healthcare technologies, irrespective of income, geography, or social status.

Humanity's well-being is interconnected, a principle highlighted in *The Promise of World Peace* (6) and reinforced in the statement that “*the welfare of any segment of humanity is inextricably bound up with the welfare of the whole*” [(7), para. 2], emphasizing the need for collective effort to ensure progress benefits all. As digital technologies increasingly shape the contours of healthcare, this principle demands renewed reflection. In a globally connected world, pandemics and health crises remind us that no population is truly isolated. Inequities in digital health preparedness and response in one region can have ripple effects worldwide. Thus, global cooperation and equitable digital infrastructure are essential.

The research contributions in this Research Topic, *Socioeconomic Inequalities in Digital Health*, illuminate both the opportunities and the tensions at the heart of this issue. A narrative review by Awosiku et al. examines the transformative potential of Digital Health Technologies (DHTs) in achieving Universal Health Coverage (UHC) in Sub-Saharan Africa. Drawing on case studies from Mali, Kenya, and Tanzania, the authors show how thoughtfully implemented DHTs can strengthen health financing, reduce structural barriers, and expand access. Yet, they also stress the need for equity-centred, privacy-conscious, and sustainable strategies, offering a roadmap for ethical innovation in settings where resources are limited and health systems are often fragmented.

Echoing these themes, Jokinen et al. present findings from Rwanda that underscore the persistent challenges faced by rehabilitation professionals in integrating digital tools. Socioeconomic barriers, infrastructural deficits, limited digital literacy, and unclear policy frameworks hinder the adoption of digital health, particularly in rural communities. Professionals report a lack of training, while patients face access barriers due to cost and connectivity. Cultural attitudes and trust in technology further influence acceptance. These insights reinforce the importance of tailoring digital health interventions to local contexts and building the capacity of both providers and users.

Crucially, digital exclusion is not confined to low-income countries. A cross-sectional study by Claudio et al. at a paediatric hospital in Canada reveals that even in high-income settings, the digital divide persists. While most caregivers had access to digital devices, 23% encountered challenges with technological familiarity and expressed concerns about data security. These findings highlight that digital readiness cannot be assumed and must be intentionally cultivated, especially among vulnerable and underserved groups. Without this, digital health risks becoming yet another layer in the architecture of inequality.

Expanding on this, Choolayil et al. offer a conceptually rich exploration of digital marginalisation. They show how older adults, rural populations, and those with low digital literacy are increasingly excluded from healthcare access as systems digitise. The authors advocate for inclusive strategies, including in-person support, hybrid models, and targeted digital training, to ensure that technological innovation does not leave the most vulnerable behind. Their work serves as a reminder that human-centred design must remain at the core of digital health.

Bell et al. add to this dialogue with a U.S.-based scoping review of interventions providing digital devices and connectivity to vulnerable populations. These efforts led to improved engagement and satisfaction with telehealth, but, as the authors caution, they remain short-term solutions. Long-term digital equity will require policy frameworks that institutionalise access and support. Moreover, the review notes a critical gap: limited evidence on whether these interventions translate into better health outcomes over time, an area that demands further study.

Finally, Dereje et al. examine the use of serious games for rehabilitation in low- and middle-income countries. These interactive tools show promise in delivering low-cost, engaging care, particularly in settings where rehabilitation services are scarce. Yet, challenges such as poor infrastructure, limited digital literacy, and the lack of culturally relevant content persist. The authors emphasise the need for locally led co-design, cross-sector collaboration, and supportive policies to harness the full potential of digital therapeutics in Low- and Middle-Income Countries (LMICs).

Together, these studies present a complex but hopeful picture. They remind us that digital health, if designed inclusively, can be a powerful tool for equity, but if left unchecked, it may reproduce the very injustices it seeks to overcome. These insights challenge us to see digital health not merely as a technical endeavour but as a profoundly human one. As *The Promise of World Peace* observes, “The process of planetization is at work, integrating peoples and cultures, reshaping institutions, and forging bonds of mutual dependence and collaboration.” When guided by compassion and foresight, digital health can become a vital part of this process, promoting dignity, justice, and well-being across all levels of society.

In the context of digital health, “planetization” means designing systems that transcend national, racial, and economic divides—systems grounded in ethics and shaped by the lived realities of those they aim to serve. It reminds us that innovation without purpose is insufficient. Only through equity, accessibility, and cultural humility can digital health fulfill its transformative potential. This means not only addressing disparities but reimagining health systems to reflect the unity of humanity.

To realise this vision, researchers must continue to interrogate both the impacts and blind spots of digital innovation; policymakers must craft frameworks that prioritise inclusion over scale; and practitioners must advocate for solutions that are as compassionate as they are cutting-edge. The task before us is not only technical—it is moral, social, and shared. Inclusive digital health policies must prioritise the marginalised, not merely enhance convenience for the already privileged. By prioritising the needs of the most vulnerable, we strengthen the fabric of health systems for all. When guided by shared purpose, justice, and the recognition of our oneness, digital health can unite humanity, fostering solidarity instead of division. This Research Topic offers both evidence and inspiration to help guide us along that vital path.
