# Correction: Editorial: Socioeconomic inequalities in digital health

**DOI:** 10.3389/fdgth.2025.1755647

**Published:** 2025-12-10

**Authors:** Lua Perimal-Lewis, Sónia Vladimira Correia, Evanthia Sakellari

**Affiliations:** 1 Flinders University, Adelaide, Australia; 2 Univerzita Palackeho v Olomouci, Olomouc, Czechia; 3 Universidade Lusófona de Lisboa, Lisboa, Portugal; 4 University of West Attica, Athens, Greece

**Keywords:** digital health, socioeconomic inequalities and health, socioeconomic inequalities in digital health, universal health coverage (UHC), digital exclusion, digital readiness, digital marginalisation, digital literacy

Affiliation Universidade Lusófona de Lisboa, Lisboa, Portugal was erroneously given as Universidade Lusofona do Porto, Porto, Portugal.

The original version of this article has been updated.

